# Supplementary material for: Long-Term Survival and Immune Response Dynamics in Melanoma Patients Undergoing TAPCells-Based Vaccination Therapy
Source: Vaccines (Basel). 2024 Mar 27;12(4):357. doi: 10.3390/vaccines12040357 (PMC11053591; doi:10.3390/vaccines12040357)
Supplement: Supplementary file 1 [file vaccines-12-00357-s001.zip › vaccines-2783041-supplementary.pdf]

Supplementary material

Supplementary Table S1. TAPCells-vaccinated melanoma patients in the clinical trials.

Code (#)	Sex	Age (*)	Stage	Met	Primary tumor	Additional treatment	Adjuvant	irAEs	DTH	Status	OS (months)
**Short-term survivors (< 36 months) (*n* = 53)**											
MT1[D]	F	41	IV	M1b	Trunk (right upper hemithorax)	Surgery	KLH + $Al(OH)_3$	No	+	D	28
MT2[D]	M	60	IV	M1b	Limb (left thigh)	Surgery	$Al(OH)_3$	Fever	-	D	16
MT3	M	35	IV	M1c	Limb (Left shoulder /armpit)	Surgery	$Al(OH)_3$ + IL-2	No	+	D	15
MT4[D]	M	39	IV	M1c	Head and neck (Left cheekbone)	Surgery	KLH	No	+	D	5
MT6	F	35	IV	M1b	Limb (Left knee)	Surgery + local RT	KLH	No	-	D	1
MT7	F	68	IV	M1c	Trunk (back)	Surgery	KLH	No	-	D	6
MT8	F	53	IV	M1a	Limb (left foot sole)	Surgery	KLH + IL-2	Low fever	-	D	11
MT11	M	64	IV	M1c	Head and neck (retro-orbital)	Surgery + RT	KLH	No	NT	D	1
MT12	M	65	IV	M1c	Limb (right foot sole)	Surgery	$Al(OH)_3$ + IL-2	No	-	D	3
MT14	M	45	IIIC	N3	Limb (left shoulder)	Surgery	KLH + IL-2	No	+	D	10
MT16	F	21	IIIC	N2c	Limb (dorsal left thigh, upper half)	Surgery	KLH	Headache, flu-like symptoms	+	D	14
MT17	M	51	IV	M1c	Head and neck (left zygomatic bone)	Surgery	KLH + IL-2	Slight nausea, low appetite	NT	D	10
MT19[D]	M	19	IV	M1c	Head and neck (right eye)	Surgery + local RT	KLH	Slight migraine	-	D	5
MT22	M	50	IV	M1c	Limb (right foot big toe)	Surgery	$Al(OH)_3$ + IL-2	No	NT	D	2
MT23[G]	M	40	IV	M1a	Head and neck (right	Surgery + RT	$Al(OH)_3$ + IL-2	No	-	D	11

					malar)						
MT24[D]	M	77	IV	N3	Limb (left suprascapular)	Surgery	$Al(OH)_3$ + IL-2	No	-	D	5
MT25[D]	M	51	IV	M1c	Trunk (right scapular, nodular venus)	Surgery + RT	KLH	No	NT	D	4
MT28	F	70	IV	M1b	Trunk (vulva)	Surgery	$Al(OH)_3$	No	+	D	7
MT29[D]	M	42	IV	M1b	Trunk (periumbilical abdomen)	Surgery	$Al(OH)_3$	No	+	D	16
MT30[D]	F	81	IV	M1c	Limb (ventral left knee)	Surgery + local RT	$Al(OH)_3$	Tiredness	+	D	27
MT32	M	59	IIIC	N3	Limb (ventral left forearm)	Surgery + RT	KLH + $Al(OH)_3$	No	+	D	10
MT34[D]	F	71	IV	M1b	Limb (subungueal big toe right foot)	Surgery	KLH	No	-	D	15
MT36[G]	F	59	IV	M1b	Limb (right dorsal calf)	Surgery + RT	KLH	No	-	D	16
MT37	M	55	IV	M1a	Trunk (right anterior cervical region)	Surgery	KLH	No	-	D	6
MT38	F	48	IV	M1c	Trunk (right dorsal subscapular hemithorax)	Surgery	KLH	Local erythema	+	D	20
MT39[D]	F	42	IV	M1a	Limb (ventral right foot)	Surgery	KLH	Local erythema	-	D	13
MT40	M	59	IV	M1c	Trunk (left dorsal thoracic with bleeding)	Surgery + local RT	KLH	No	-	D	7
MT41[D]	M	60	IV	M1b	Head and neck (right retroauricular)	Surgery	KLH	No	+	D	4
MT42	M	53	IV	M1b	Trunk (right suprapubic inguinal)	Surgery + RT + Temozolomide	KLH	No	-	D	3
MT43[D]	F	71	IV	M1a	Limb (right heel)	Surgery	KLH	No	+	D	21
MT44	F	25	IV	M1a	Trunk (left	Surgery +	KLH	No	+	D	14

					scapula)	local RT					
MT46[G]	M	69	IV	M1b	Trunk (back)	None	KLH	No	-	D	8
MT48[D]	F	44	IV	M1c	Limb (left forearm)	None	KLH	No	+	D	9
MT50[D]	F	52	IV	M1c	Limb (left foot sole)	RT + temozolomi de	KLH	No	+	D	3
MT53[D]	F	72	IV	M1c	Limb (left ankle)	None	KLH	No	-	D	5
MT60[D]	M	42	IV	M1a	Limb (right leg)	IL2 + RT	KLH	No	+	D	26
**MT61[G]**	M	80	IV	M1c	Trunk (urethral meatus)	CTX	KLH	No	-	D	11
**MT64**	F	45	IV	M1c	Head and neck (pharynx)	CTX	KLH	Local erythema	-	D	15
**MT65[G]**	F	42	IV	M1c	Head and neck (scalp)	CTX	KLH	No	-	D	6
**MT72**	M	42	IV	M1c	Trunk (dorsal)	None	KLH	No	-	D	11
**MT79[D]**	F	64	IV	M1c	Trunk (left flank)	None	KLH	No	-	D	9
**MT83[D]**	M	57	IIIC	N3	Limb (right hand)	None	KLH	No	-	D	17
MT88	F	70	IV	M1c	Head and neck (uveal melanoma)	RT + TAM + DTIC	KLH	No	+	D	8
**MT96[D]**	M	46	IIIA	N2a	Trunk (dorsal)	None	$Al(OH)_3$	Local erythema	+	D	31
**MT114[D]**	M	47	IV	M1c	Trunk (thoracic wall)	RT + IFN-α	$Al(OH)_3$	No	-	D	11
MT118[D]	F	40	IV	M1a	Limb (left shoulder)	ND	KLH	Dermatiti s	+	D	28
MT120[D]	M	36	IV	M1b	Trunk (dorsal)	DTIC	KLH	Local erythema	+	D	12
**MT122[D]**	M	23	IIIC	ND	Limb (left leg)	None	KLH	Local erythema	+	D	30
**MT123[D]**	M	44	IV	M1c	Trunk (left flank)	RT	KLH	No	-	D	7
**MT125[D]**	F	54	IIIC	N3	Limb (right leg)	None	KLH	Urticaria reaction to KLH	+	D	13

MT127[D]	F	37	IV	M1b	Limb (left foot)	None	KLH	Local erythema	+	D	12
MT135	M	72	IV	M1a	Head and neck (left retroauricular)	None	KLH	No	-	D	16
MT136[D]	F	49	IV	M1b	Limb (left gluteus)	None	KLH	No	-	D	4
**Long-term survivors (≥ 36 months) (*n* = 33)**											
MT5	F	24	IIIC	N3	Limb (left shoulder)	Surgery	KLH	No	+	R	247
MT9	M	62	IV	M1a	Head and neck (scalp)	Surgery + IL-2	KLH + IL-2	No	+	R	241
MT10	F	57	IIIC	N3	Unknown	IL-2	KLH + IL-2 + $Al(OH)_3$	No	+	R	240
MT13	F	38	IIIC	N3	Head and neck (left cheekbone)	Local RT + IL-2	KLH + IL-2	No	+	R	235
MT15	M	30	IV	M1a	Trunk (abdomen skin)	Surgery + IL-2	KLH + IL-2	Flu-like symptoms	+	D	40
MT18	F	63	IV	M1b	Limb (left ankle exterior side)	Surgery	KLH + $Al(OH)_3$	No	+	D	36
MT20[G]	F	51	IV	M1c	Head and neck (uveal melanoma)	Local RT	KLH	No	+	D	41
MT21[D]	F	61	IV	M1a	Head and neck (scalp)	Surgery + local RT	KLH + $Al(OH)_3$	No	+	D	133
MT26[D]	M	54	IV	M1a	Limb (ventral left thigh)	Surgery + IL-2	$Al(OH)_3$ + IL-2	No	+	R	221
MT27	M	47	IV	M1b	Trunk (abdominal wall)	None	KLH	Slight migraine	+	R	221
MT31[D]	F	42	IV	M1a	Head and neck (right eyelid)	Surgery + local RT	KLH + $Al(OH)_3$	Flu-like symptoms	-	D	62
MT33[D]	M	44	IIIC	N3	Limb (right calf)	None	KLH	No	+	R	215
MT35[D]	F	66	IV	M1a	Limb (left dorsal calf)	IFN-α + surgery	KLH	No	-	D	119
MT45[G]	M	19	IV	M1a	Unknown	Surgery + local RT	KLH	No	+	D	62

MT49[D]	M	54	IV	M1a	Limb (left foot sole)	None	KLH	No	+	R	187
MT58[D]	F	36	IV	M1a	Limb (left ankle)	RT	KLH	No	+	R	188
**MT62**	F	40	IV	M1a	Trunk (dorsal)	CTX	KLH	No	+	R	184
**MT66[D]**	M	54	IIIC	ND	Trunk (abdominal wall)	RT + DTIC	KLH	No	+	D	40
MT76[D]	F	29	IV	M1b	Limb (left foot)	RT	KLH	No	+	D	53
**MT80**	M	74	IV	M1c	Head and neck (palate)	ND	KLH	No	+	D	152
**MT84[D]**	M	47	IIIC	N3	Limb (right hand)	None	KLH	No	+	D	66
**MT91[D]**	M	70	IV	M1c	Head and neck (scalp)	None	$Al(OH)_3$	Local erythema	+	D	51
MT94[D]	F	48	IV	M1c	Head and neck (uveal melanoma)	RT	$Al(OH)_3$	Local erythema	+	R	175
**MT101[D]**	M	41	IV	M1a	Limb (right leg)	None	KLH	Vitiligo	+	D	128
**MT102[D]**	F	46	IV	M1b	Limb (right thigh)	DTIC + Ip	KLH	No	+	D	36
**MT103[D]**	F	47	IIIB	N2b	Limb (left foot)	None	$Al(OH)_3$	Flu-like syndrom e	+	D	64
**MT105[D]**	F	56	IIIA	N2a	Limb (right leg)	None	$Al(OH)_3$	No	-	R	174
MT112[D]	M	67	IIIA	N2a	Trunk (dorsal)	None	$Al(OH)_3$	Local erythema	+	R	172
MT115[D]	F	46	IB	NA	Limb (left shoulder)	None	$Al(OH)_3$	No	+	R	171
MT126[D]	F	39	IB	NA	Limb (left axillary)	None	KLH	No	+	D	41
**MT128[D]**	F	34	IIIB	N2c	Limb (right shoulder)	RT	KLH	Local erythema	+	R	167
MT132[D]	M	28	IV	M1c	Head and neck (uveal melanoma)	DTIC	KLH	Vitiligo	-	D	44
MT133[D]	F	30	IIIB	N2a	Trunk (thorax)	None	KLH	No	-	R	166

CTX: cyclophosphamide; D: deceased; DTH: delayed-type hypersensitivity against TRIMEL; DTIC: dacarbazine; TAM: Tamoxifen; Ip: Ipimilumab F: female; IL: interleukin; IFN: interferon; irAEs: immune-related adverse events; Ip: Ipilimumab; KLH: keyhole limpet hemocyanin; NA: not applicable; ND: not determined; NT: not tested; M: male; Met:

metastasis (N2a: spread to 2 to 3 nearby lymph nodes, micrometastases; N2b: spread to 2 to 3 nearby lymph nodes, macrometastases; N2c: spread to 2 to 3 nearby lymph nodes, in transit; N3: four or more lymph nodes, metastatic or matted, or in-transit met(s)/satellite(s) with metastatic lymph node(s); M1a: in transit skin metastases; M1b: lung metastasis; M1c: metastases in organs other than lungs); OS: overall survival time; P: progressor; R: in remission; RT: radiotherapy; TAM: tamoxifen. (*) Age at treatment initiation. (#) D and G upper letters indicate D/D or D/G-G/G TLR4 genotypes [13]. In red are depicted patients with data for Th1 and Th17 T cell populations and for serum IL-17 and transforming growth factor (TGF)-β [9]. The underline indicates patients with data for CLEC2D, CXCR4, or CD32 expression in peripheral blood leukocytes [10]. Patients MT3, MT8, MT10, and MT34 were revaccinated with a second round of TAPCells one year after the first cycle.

Supplementary Table S2. Characteristics of the cohort of advanced melanoma patients treated with TAPCells in a compassionate use setting.

Code (*)	Sex	Age	Stage	Met	Primary tumor	Additional treatment	DTH	Status	OS (months)
**Short-term survivors (< 36 months) (*n* = 17)**									
MT140	M	45	IV	M1b	Limb (right foot)	None	-	D	9
MT141	M	55	IV	M1c	Limb (knee)	RT	-	D	21
MT144^{G}	F	26	IIIA	N2a	Limb (right leg)	DTIC + IFN	+	D	14
MT147^{G}	F	33	IV	M1b	Limb (left leg)	RT	+	D	13
MT148	M	44	IV	M1a	Limb (left leg)	None	+	D	22
MT151	M	62	IV	M1c	Limb (right leg)	None	-	D	3
MT154	M	39	IV	M1c	Unknown	None	+	D	12
MT158	M	55	IV	M1a	Limb (right shoulder)	Surgery + RT	-	D	10
MT164	F	66	IV	M1a	Unknown	DITC + CP + VBL	-	D	15
MT166	F	53	IV	M1b	Head and neck (facial chin)	Surgery + RT	+	D	15
MT167	F	37	IV	M1b	Trunk (upper hemithorax)	Surgery + RT + temodal	+	D	23
MT168	F	51	IV	M1c	Limb (left foot sole)	Surgery + RT	-	D	4
MT171	M	28	IV	M1c	Head and neck (uveal melanoma)	RT + DTIC	-	D	7
MT175	M	64	IV	M1b	Limb (left arm)	Surgery	+	D	34
MT183	M	70	IV	M1b	Limb (left shoulder)	Surgery + RT	-	D	8

MT198	F	68	IV	M1b	Limb (right foot)	None	-	D	18
MT199	F	64	IV	M1b	Limb (left heel)	None	+	D	1
**Long-term survivors (≥ 36 months) (*n* = 7)**									
MT146	F	77	IIIA	N2a	Limb (right leg)	None	+	D	44
MT156	F	61	IV	M1c	Trunk (vaginal mucosa)	Surgery	+	R	157
MT159	M	60	IV	M1a	Trunk (right groin)	Surgery	+	R	156
MT160	M	51	IV	M1a	Head and neck (left cheek)	DITC + CP + VBL	+	R	155
MT180	M	32	IIIC	N2c	Trunk (right dorsal)	Surgery + local RT	+	R	149
MT184	F	36	IV	M1b	Limb (armpit)	Surgery	+	D	53
MT193	F	53	IV	M1b	Unknown	None	+	R	146

CP: cisplatin; D: deceased; DITC: dacarbazine; DTH: delayed-type hypersensitivity against TRIMEL; F: female; IFN: interferon; M: male; Met: metastasis; N: with metastases in lymph nodes; M1a: in transit skin metastases; M1b: lung metastasis; M1c: metastases in organs other than lungs; OS: overall survival time; R: in remission; RT: radiotherapy; VBL: vinblastine. (*) G upper letter indicates D/G or G/G TLR4 genotypes [13]. Underlined are depicted patients with data for CLEC2D, CXCR4 expression, or CD32 in peripheral blood leukocytes [10]. All these patients were treated with keyhole limpet hemocyanin (KLH) as an adjuvant. There is no data about adverse effects for this cohort.

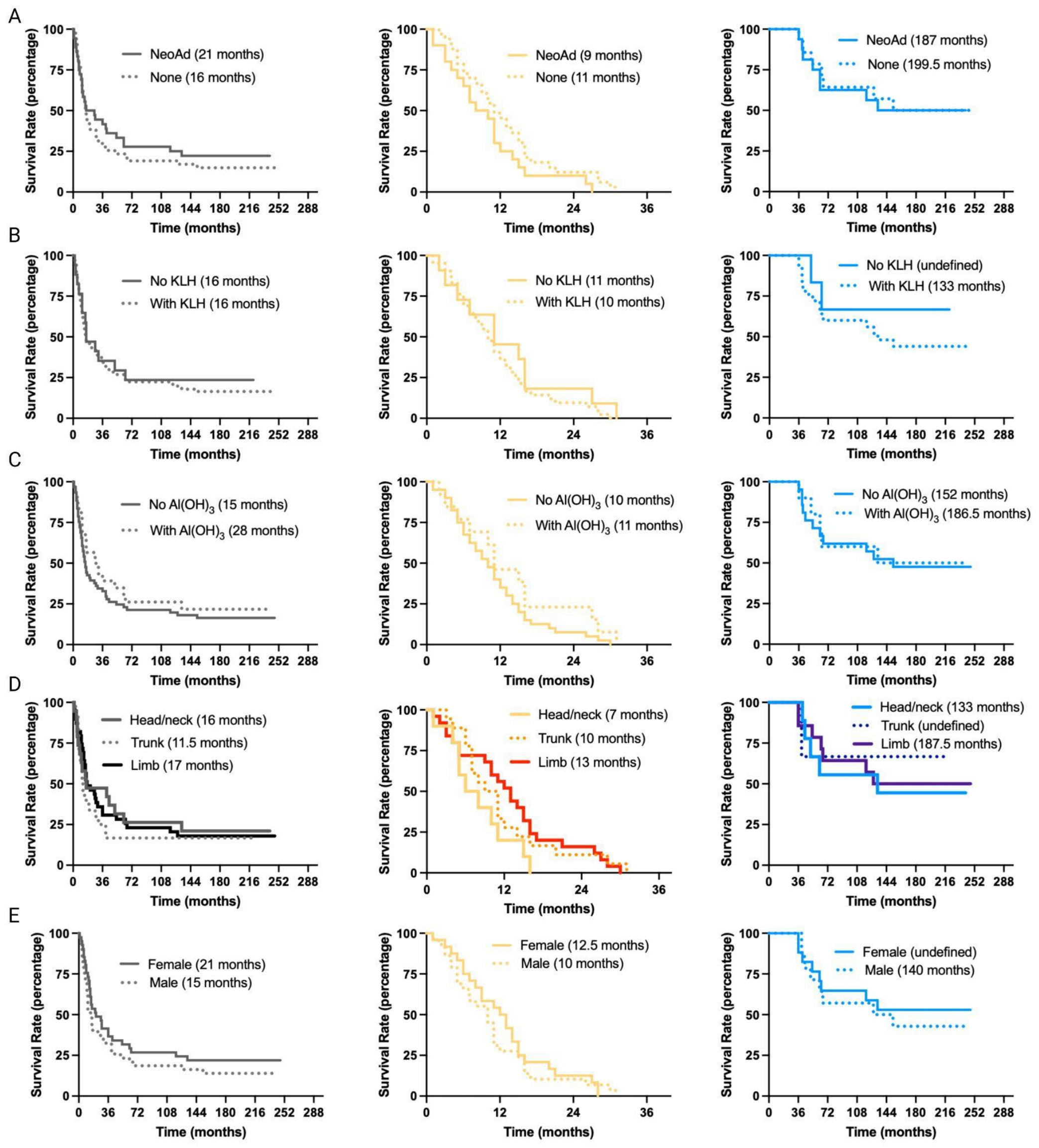

Supplementary Figure S1. Overall survival outcomes in short-term and long-term survivors of advanced melanoma patients treated with TAPCells in clinical trial settings. (A-E) Kaplan–Meier post–TAPCells treatment overall survival estimation for advanced melanoma patients (left panels: complete cohort; middle panels: short-term survivors; and right panels: long-term survivors) according to use of neoadjuvant (A; NeoAd), use of KLH (B) use of $Al(OH)_3$ (C), primary tumor location (D), and sex (E). The median overall survival times in months are shown.

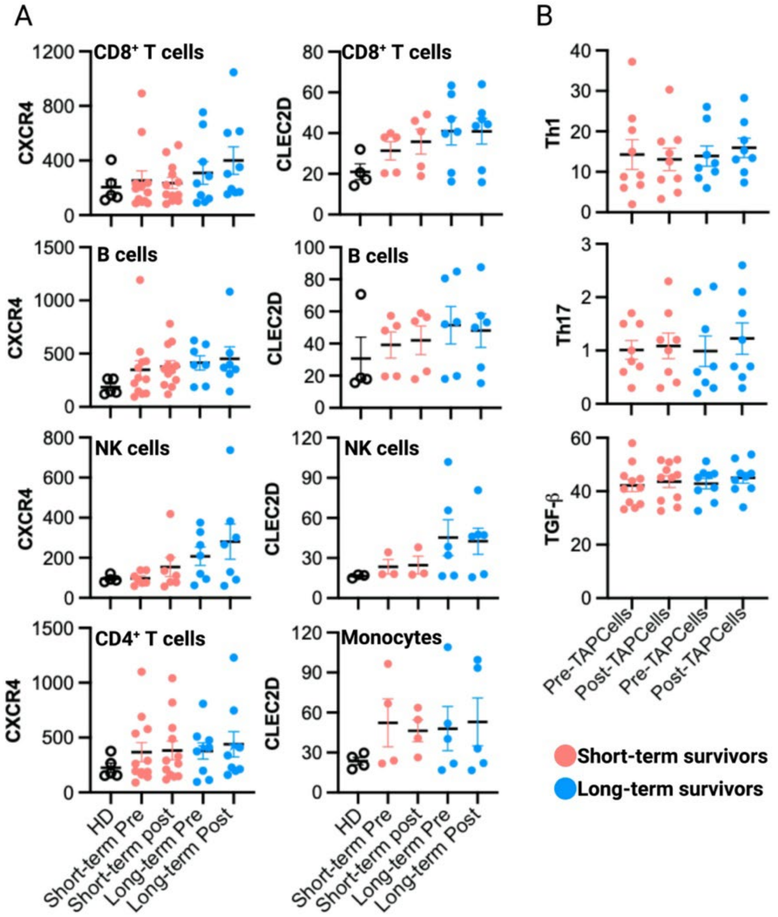

Supplementary Figure S2. Immunological aspects associated with long-term overall survival in MM patients treated with TAPCells. (A) Cryopreserved peripheral blood mononuclear cells from healthy donors (HD; *n* = 4–5), short- (*n* = 3–12), and long-term (*n* = 5–9) survivor patients, at the beginning (pre-TAPCells) and at the end (post-TAPCells) of the immunization protocol, were analyzed for CLEC2D and CXCR4 surface expression in monocytes, $CD4^+$ T cells, $CD8^+$ T cells, B cells, and natural killer (NK) cells. Each data point represents one patient sample. The graphs show the mean fluorescence intensity of each marker in the corresponding cell type. (B) The percentage of $CD4^+IFN\text{-}\gamma^+$ (Th1) and $CD4^+IL\text{-}17^+$ (Th17) T cells and the serum levels of TGF-β (ng/mL) for short- (*n* = 10) and long-term (*n* = 4) survivors, at the beginning (pre-TAPCells) and at the end (post-TAPCells) of immunization protocol were analyzed by flow cytometry and ELISA, respectively.
